# Physical symptoms in very young children assessed for sexual abuse: a mixed method analysis from the ASAC study

**DOI:** 10.1007/s00431-017-2996-7

**Published:** 2017-08-26

**Authors:** Thekla F. Vrolijk-Bosschaart, Sonja N. Brilleslijper-Kater, Guy A. Widdershoven, Arianne (Rian) H. Teeuw, Eva Verlinden, Yolande Voskes, Esther M. van Duin, Arnoud P. Verhoeff, Marc A. Benninga, Ramón J. L. Lindauer

**Affiliations:** 1 Department of Social Pediatrics, Child Abuse and Neglect Team, Emma Children’s Hospital, Academic Medical Center Amsterdam, AMC, Meibergdreef 9 (h7-288), 1105 AZ Amsterdam, the Netherlands; 2 Department of Medical Humanities, VU University Medical Center, Amsterdam, the Netherlands; 3 Department of Epidemiology, Health Promotion and Healthcare Innovation, Public Health Services, Amsterdam, the Netherlands; 4 Department of Child and Adolescent Psychiatry, Academic Medical Center, Amsterdam, the Netherlands; 5 Department of Sociology, University of Amsterdam, Amsterdam, the Netherlands; 6 Department of Pediatric Gastroenterology, Emma Children’s Hospital, Academic Medical Center, Amsterdam, the Netherlands; 7 De Bascule, Academic Center for Child and Adolescent Psychiatry, Amsterdam, the Netherlands

**Keywords:** Child sexual abuse, Recognition, Diagnosis, Physical complaints, Anogenital examination

## Abstract

So far, a recognizable pattern of clinical symptoms for child sexual abuse (CSA), especially in young male children, is lacking. To improve early recognition of CSA, we reviewed physical complaints, physical examination, and tests on sexually transmitted infections (STIs) in confirmed victims (predominantly preschool boys) of CSA from the Amsterdam sexual abuse case (ASAC). We retrospectively analyzed the outcomes of the primary assessment using mixed methods: descriptive analysis of physical complaints, physical exams, and STI tests from medical files and a qualitative analysis on expert’s interpretations of physical complaints and children’s behavior during physical examination. We included 54 confirmed CSA victims, median age 3.2 (0–6) years, 43 boys (80%), and 11 girls (20%). Physical complaints were reported in 50%, of which gastrointestinal and anogenital complaints were most common. None of the children showed CSA-specific genital signs at physical examination. Most prominent finding during physical examination was a deviant behavioral response (anxiety, withdrawal, too outgoing) in 15 children (28%), especially in children who experienced anal/vaginal penetration. Testing for STIs was negative.

*Conclusion*: Physical complaints and physical signs at examinations were non-specific for CSA. Deviant behavioral reactions during physical examination were the most prominent finding. Precise observation of a child’s behavior during physical examination is needed.

**Table:** 

**What is known** • *Child sexual abuse (CSA) affects many children on both the short and the long term but remains unrecognized in most cases.* *• So far, there is a lack of studies on symptom patterns of CSA in male, preschool children.*
**What is new** • *None of the children showed CSA-specific findings at physical and anogenital examination; STIs were not found in the confirmed victims of CSA.* *• The most prominent finding was the deviant behavioral response of the children examined, especially in children who experienced anal/vaginal penetration; therefore, precise observation of a child’s behavior during physical examination is a crucial part of the evaluation of suspected CSA.*

**Electronic supplementary material:**

The online version of this article (10.1007/s00431-017-2996-7) contains supplementary material, which is available to authorized users.

## Introduction

The prevalence of child sexual abuse (CSA) is estimated between 4 and 31% [6] with girls more likely to become CSA victims than boys [6, 28]. Both short- and long-term consequences of CSA can be serious, varying from psychosocial problems (such as depression, post-traumatic stress, or substance abuse) to physical health problems (such as acute injuries and functional somatic syndromes) [3, 8, 18]. Therefore, it is necessary to diagnose CSA at an early stage in order to stop the abuse and to prevent or treat consequences.

However, recognizing CSA, especially in young children, remains difficult. As shown in discrepancies between informant and self-reported abuse, CSA in children remains unrecognized in most cases [28]. Unfortunately, most children do not disclose until adult life, let alone at preschool age [20, 22].

Psychosocial symptoms, such as learning and behavioral problems [16], are often nonspecific for CSA and are absent in about 30% of the children [11]. Age inappropriate sexual behavior in children can be useful as discriminating variable between sexual abused and non-abused children [5, 9]. However, no specific sexual behavior is indicative of CSA [13]. Other explanations for this behavior, such as physical abuse, family violence, and other types of maltreatment, are also plausible [10, 13].

Other indicators for CSA can be physical complaints, findings at genital examinations, and sexually transmitted infections (STIs). Among adolescents, functional somatic symptoms (FSS) are associated with experienced CSA [7]. Yet, we do not know whether this is applicable to preschool children. Physical signs at examination, specific for CSA, are found in a small minority of cases, 4 to 5%, when examined over 72 h after the last abuse [2, 24]. Recently, the Royal College of Pediatrics and Child Health (RCPCH) urgently addressed the need for primary research studies on physical signs of CSA and male genital injury in particular, with attention to the security of diagnosis, since sufficient data in this area are lacking [24].

To improve early recognition of CSA, the current study reviews the physical complaints and the results of the physical examination and tests for STIs in very young, predominantly male children who were confirmed victims of CSA in the Amsterdam sexual abuse case [17].

## Methods

### Study setting

In 2010, a day-care center employee and babysitter in Amsterdam sexually abused dozens of young children. Many very young children, mostly boys, were considered possible victims in what was called the Amsterdam sexual abuse case (ASAC)—the largest confirmed CSA case in history. The ASAC is a unique case, due to its large scale, the predominance of young boys, the confessing and convicted perpetrator, the high level of evidence, and detailed documentation available about the abuse. Child pornographic images were decrypted in police investigations, and the employee admitted CSA of 87 children. Parents of 20 children decided against pressing charges, and the day-care worker was convicted for abusing 67 children.

In the Emma Children’s hospital of the Academic Medical Center in Amsterdam (AMC), an emergency outpatient department (OPD) was set up to examine 130 possible victims of CSA involved in the ASAC (of whom 54 confirmed victims). Children were referred to the AMC if there was a strong suspicion of CSA: the child was currently (or previously) visiting a day-care center where the perpetrator worked, or the perpetrator currently (or previously) worked as a babysitter at the child’s home; or when a child was a confirmed victim of CSA (identification of the child via encrypted pornographic images detected by the police or the perpetrator gave a confession).

### Procedure

#### Medical assessment

Five multidisciplinary teams composed of a pediatrician, a social worker, and a child behavioral specialist evaluated all the presented cases. The pediatricians performed a semi-structured medical interview (using a medical topic list, appendix 1) with parents and their children and a full top–toe physical examination, including anogenital examination, which was recorded photographically. No standardized questionnaires were used during the OPD assessment. A child behavioral specialist observed all children during the consults, also during the physical exams.

Children were tested for STIs (PCR gonorrhea, chlamydia trachomatis; serology for human immunodeficiency virus (HIV), hepatitis B and C (Hep. B and C), and herpes simplex virus (HSV)).

#### Police reports

Information about the nature and severity of the abuse was collected from police reports and indictments. One investigator (EV) was authorized to read the declarations of the perpetrator and documented for each child whether there was a confession, pornographic photographs, or videos or none.

### Study design

We performed a mixed method study combining the following: (1) a cross-sectional study focusing on the physical complaints, physical exams, and STI-tests in confirmed victims of CSA and (2) a qualitative approach on interpreting physical complaints and children’s behavior during the physical examination written in medical files.

For the cross-sectional study, we only included confirmed victims of CSA, meaning there was a confession of the perpetrator and/or the child was identified on decrypted pornographic images. For the qualitative analysis, both the confirmed victims and children seen for strong suspicions of CSA were included to prevent bias.

### Data extraction and analysis

Data were extracted from the original medical files of the confirmed victims of the ASAC by two investigators (TFVB and SNBK). Physical complaints, including gastro-intestinal, anogenital or urological complaints, anogenital blood loss, skin problems and other physical health problems, deviant physical exams (general pediatric examinations, anogenital examinations, and behavioral observations during the exam), and positive results of STI-tests, were reported in the medical file.

Statistical analysis software (SPSS v.24) was used for comparative analysis, Fisher exact test (or Chi-square test where appropriate). Significance was defined as *p* ≤ 0.05.

### Interpretation of reported findings

For the interpretation of the anogenital findings, we used the latest recommendations of the RCPCH guideline [24] (Table 1). According to the Center for Disease Control and Prevention (CDC) guidelines, positive STI-tests for gonorrhea, syphilis, chlamydia trachomatis, and HIV are diagnostic for CSA if perinatal and vertical transmission is excluded (and in the case of HIV transmission via blood products). Genital herpes is considered highly suspicious for CSA unless a clear history of auto-inoculation exists [1].

**Table 1:** Anogenital signs of CSA [39]

	Genital signs of CSA in prepubertal girls and boys	Anal signs of CSA
Non-specific signs	Erythema, hymenal bumbs/mounds	Perianal venous congestion, perianal midline tags
Insufficient evidence	Edema	Anal/perianal erythema
Limited evidence, CSA should be considered	Vaginal discharge, vaginal foreign body	
Sign of trauma	Bruising, abrasions, genital/hymenal lacerations Genital injuries, predominantly to the penis^a^	Dynamic anal dilatation or total dilatation of both internal and external sphincter in the absence of stool Anal/perianal bruising, anal laceration^a^
Healed trauma	Hymenal transections	Perianal scars and tags outside the midline

^a^Though evidence is limited

To our knowledge, standardized methods to interpret the physical complaints and behavioral reactions during physical examinations are lacking; therefore, we asked experts to interpret the written findings.

We selected five experts on CSA based on their expertise in this field (two child behavioral scientists (EV and SNBK), two pediatricians (AHT and LvdB), and one child psychiatrist (RJLL)) (further called “experts”). All experts cooperated on voluntary basis.

The experts were asked to evaluate anonymized summaries of the included cases (*n* = 54 confirmed victims) and of children seen for strong suspicions of CSA without legal evidence for CSA (no confession by the perpetrator or identification on pornographic images (*n* = 71)). Case summaries contained information about age and sex of the child, the time period of exposure to the perpetrator, medical history, physical exam, psychosocial problems, and child interviews. Data from the police investigation were withheld to prevent bias.

The experts scored the physical complaints and physical examination independently and gave an overall conclusion on the case as a whole. Scores by experts varied between 1 and 4, meaning 1 not worrisome, 2 somewhat worrisome, 3 worrisome, 4 very worrisome.

Scores of the experts were evaluated on basis of consensus. There was consensus if all 5 experts scored a case either as not worrisome–somewhat worrisome (1–2) or all experts scored a case as worrisome–very worrisome (3–4). If 1 of all 5 experts scored differently, no consensus was reached and the case was discussed during focus group discussions (FGDs).

Two FGDs were organized to discuss the cases where no consensus was reached. The FGDs were prepared with the help of an independent researcher specialized in qualitative research (YV). The FGDs were video-recorded. Data were analyzed using inductive content analysis as described in our previous study .

## Results

A total of 130 children visited the OPD between December 2010 and January 2012 and were evaluated for (strong suspicions of) CSA, of whom 54 were confirmed victims, who were all included. The perpetrator was convicted for sexually abusing 87 children of whom 54 were evaluated in the AMC. The median age of the children was 3.2 (0–6) years, of whom 43 boys (80%, median age 3.2 years) and 11 girls (20%, median age 2.8 years). The perpetrator confessed CSA for all included 54 children; additionally, the abuse was also confirmed by encrypted pornographic images in 27 children (50%). Table 2 summarizes the nature of abuse. Seventeen children were victims of anal or vaginal penetration, 29 children were victims of oral copulation.

**Table 2:** Demographics and abuse specific information based on police reports

		Total
		*N* = 54 (*N* = 43, 80% male)
Age (median, min-max)		3.2 (0–6) years
Pornographic images encrypted		*N* = 27 (50%)
Nature CSA^a^	Exposure of genitals to the child	*N* = 49 (91%)
	Ejaculate on the child	*N* = 38 (70%)
	Fondling	*N* = 53 (98%)
	Licking the child	*N* = 26 (48%)
	Oral copulation	*N* = 29 (54%)
	Digital or penile penetration (or with a sex toy) of anus or vagina.	*N* = 17 (31%)
Frequency of CSA^b^	Less than 5 times	*N* = 30 (55%)
	More than 5 times	*N* = 11 (20%)
	More than 10 times	*N* = 9 (17%)
	Unclear	*N* = 4 (7%)
Mean estimated delay between last abuse and assessment in years (SD)^b^		1.2 (0.97)

^a^Most children were victims of various types/natures of CSA; therefore, the total number exceeds the total amount of children involved

^b^According to the perpetrator’s testimonies

Based on the perpetrator’s testimonies, 18 children (33%) were abused once or twice, nine children (17%) were abused more than 10 times. In 13 children, the last abuse happened in the past year (2010), whereas in the other 37 children, the last abuse happened between 2007 and 2009.

### Physical complaints

In half of the children (51%), no physical complaints were reported. Parents of 26 children (49%) reported one or more physical complaints (maximum was four).

Table 3 summarizes the gastrointestinal and anogenital complaints. Constipation and abdominal pain (AP) were the most frequent reported gastrointestinal symptoms. Parents reported problems like “at times the child reports AP, regular complaints of AP; the child tends towards constipation, during that time he was constipated occasionally.”

**Table 3:** Physical complaints reported in medical files

		Total
		*N* = 26 (49%) (*N* = 21, 81% males)
Number of physical complaints reported	1–2	*N* = 21 (39%)
	3–4	*N* = 6 (11%)
Gastro-intestinal complaints		*N* = 12 (22%)
		Constipation *N* = 4
		Abdominal pain (AP) *N* = 5
		Constipation and abdominal pain *N* = 1
		Other GI complaints *N* = 2
Anogenital or urological complaints		*N* = 20 (37%)
		Genital skin lesions *N* = 11
		Genital pain *N* = 3
		Genital skin lesions and pain *N* = 3
		Other *N* = 3
Blood in diaper or anogenital area		*N* = 2 (4%)
Skin lesions (non-anogenital)		*N* = 4 (7%)
Other physical complaints		*N* = 1 (2%)

Anogenital complaints, such as genital skin lesions, genital pain, and other anogenital complaints, were reported in 20 (37%) children.

According to the experts, none of the above reported physical complaints were specific for CSA. In two children (4%), parents reported incidental blood in the diaper or anogenital area. In one of these children, constipation was reported; in the other case, the blood loss was an isolated problem with no clear origin. According to our experts, anogenital injuries are indicative for any kind of trauma including accidental trauma and CSA.

## Physical examination

Physical examination was performed in all children, including anogenital examination which was recorded photographically for detailed evaluation (Table 4). The medical files of 29 children (54%) reported no abnormal findings, and in five children (9%), there were general pediatric findings reported, such as a cardiac murmur or palpable lymph nodes, not related to the anogenital area or sexual abuse. In 8 cases (15%), non-specific findings related to the anogenital area, such as diaper rashes and anogenital erythema, were reported. In 15 children (28%), the clinician reported an abnormal behavior during examination possibly related to CSA. In one child, information about physical examination was missing.

**Table 4:** Physical exam

	Total (*N* = 54)	Boys	Girls
No abnormalities noted	*N* = 29 (54%)	*N* = 22	*N* = 7
Yes, but not related to anogenital area or CSA	*N* = 5 (9%) *(cardiac murmur, dry skin on the extremities, palpable lymph nodes, hematoma shinbone, upper airway infection, depigmentation on torso)*	*N* = 3	*N* = 2
Yes, related to anogenital area but non-specific for CSA	*N* = 8 (15%) *(diaper rash which looks like candida, physiological phimosis, erythema around anus, erythema labia majora)*	*N* = 6	*N* = 2
Yes, signs of (healed) trauma, possible related to CSA	*N* = 0 (0%)	*N* = 0	*N* = 0
Yes, clinician reported behaviors during examination possible worrisome for CSA	*N* = 15 (28%)* *(child appears timid, child is a bit anxious, very anxious child and therefore not possible to examine, normal behavior up till genital examination than he panics, anxious and clingy therefore not fully able to perform the examination, resistant at the beginning but eventually able to examine him, shuts down when anus is examined (puts face into toy) not able to get contact at that moment, very anxious boy who does not want to undress and show his buttocks, did not seem to like it to be examined, child shuts down and becomes angry when physical examination is explained, a bit resistant and buzzy child who seems to know the knee—chest position without explanation)*	*N* = 15	*N* = 0
Missing	*N* = 1 (2%)	*N* = 1	*N* = 0

### Behavior during physical examination

In 28% of the children, deviant behavior was observed during physical examination. Fear and anxiety were reported in five children. The anxiety could be obstructing the exam in various degrees—“[boy, 4] at first he refused to take of his cloths, with a lot of effort we finally succeeded. “—“[boy, 3.5] watchful, extremely anxious. Clings to his parents, eventually able to inspect skin, mouth and genitals because the child was not approachable.” Our experts rated this reaction at physical examination as inappropriate because the level of panic and alertness in this patient was too high as would be expected in a child this age.

Fear and anxiety were noticed in a number of children during general physical exam—“[boy, 4] a very anxious boy who was afraid to undress and said ‘I do not want them to see my buttocks’” —“[boy, 2] Physical examination went well until the diaper was taken off, from that moment he panicked and as soon as the genital examination was over he became calm again.” This example clearly shows a sudden change in behavior and apparent anxiety related to the genital exam. This was considered worrisome, clearly age inappropriate, and a possible sign of CSA.

Another worrisome aspect was when a child’s appearance changed from open-minded to silent and withdrawn during anogenital examination—“[boy, 3 (almost 4)] during the examination of his anus he clearly appeared withdrawn, he putted his face in his stuffed animal and the physician was not able to connect with him.”

The following example underlines the opposite of resistance for the examination which raised concerns as well—“[boy, 6] at first resistant towards the genital examination but after some explanation he was cooperative; he immediately knew how to lie in knee-chest position without any explanation.”

We found no significant differences for the presence or absence of deviant behaviors during the physical examination and experience of oral copulation, frequency of abuse (less than 5 times versus more than 5 times), mean age at time of assessment at the OPD, mean estimated age at start of CSA, ending of CSA and delay between last abuse and assessment at the OPD. In children who experienced anal/vaginal penetration, clinicians reported significant more behavioral reactions compared to children who did not experience anal/vaginal penetration (47 versus 19%, p.041) (Table 5).

**Table 5:** Association between behavioral reactions and abuse specific information

		Behavioral reactions noted	No behavioral reactions noted	Significance
Frequency of CSA^a, d^	< 5 times	8 (28%)	21 (72%)	0.55
	> 5 times	5 (25%)	15 (75%)	
Nature of CSA^a^	Oral copulation	8 (29%)	20 (71%)	0.60
	No oral copulation	7 (28%)	18 (72%)	
	Vaginal/anal penetration	8 (47%)	9 (53%)	0.041
	No vaginal/anal penetration	7 (19%)	29 (81%)	
Mean age at assessment in years (SD)^b^		3.3 (1.54)	3.1 (1.31)	0.57
Mean age at start of CSA in years (SD)^b, c^		1.3 (0.88)	1.5 (0.93)	0.59
Mean age at ending of CSA in years (SD)^b, c^		2.1 (1.15)	2.0 (1.12)	0.49
Mean delay between last CSA and assessment in years (SD)^b^, ^c^		1.2 (0.99)	1.2 (0.99)	0.54

^a^Using Fisher exact test

^b^Using independent samples *t* test

^c^Estimation based on perpetrator’s testimony

^d^Missing data = 2

### Laboratory findings

None of the children tested for STIs were found positive for HIV, hepatitis B and C, syphilis, chlamydia, or gonorrhea. Ten children were found to be IgG positive herpes simplex virus, but in none of the children, genital herpes was reported.

## Discussion

The ASAC study [17] represents a unique case of CSA, including very young children, all confirmed as victims of CSA by police reports. Half of these children presented with one or more physical complaints reported by their parents. Gastrointestinal symptoms such as abdominal pain and constipation and anogenital symptoms such as skin lesions or genital pain were described most frequently. Although none of the children showed CSA-specific genital abnormalities at physical examination, deviant behavioral response (anxiety, withdrawal, too outgoing) before or during physical examination was observed in almost one third of the children. Extensive laboratory testing revealed no STIs in all children.

### Physical complaints

Abdominal pain and constipation were reported in 22% of the confirmed CSA victims. These findings, however, were considered as nonspecific for CSA, since the prevalence of abdominal pain-related functional gastrointestinal disorders and constipation in children aged 4–18 years is similar and ranges from 1.6 to 41.2% and 0.7 to 29.6%, respectively [14, 21]. Both abdominal pain and constipation are associated with stressful life events [14, 23].

In our sample, many parents reported anogenital erythema in their child. Anogenital erythema is, however, nonspecific for CSA. Possible causes of anogenital erythema include acute trauma from any cause, infection, dermatological condition, and excessive or poor hygiene [24].

The origin of genital blood loss in prepubertal girls is often unknown; it might be genital, or from the skin, urinary tract, or anus. The RCPCH guideline advices that children with possible genital blood loss, without obvious cause, should be evaluated for CSA by specialists [24]. Supportive literature is, however, lacking. For the two cases in our sample, we do not know the origin of the blood loss in one case; in the other case, both the constipation as the anal penetration could have been the cause of recto anal blood loss.

### Laboratory findings

After the OPD assessments were performed, it became known that the perpetrator tested positive on HSV-IgG and negative on the other STIs. In our sample, 10 children were tested positive on HSV-IgG, but none reported genital herpes. Diagnosis of HSV infection is usually made by sampling an active lesion and testing it for the presence of the virus by PCR, direct fluorescent antibody methods, or viral culture [4]. As none of the children reported genital herpes infections and none presented with HSV lesions at time of evaluation, sampling could not be done. Vertical and non-sexual transmission in these children could not be excluded.

The evidence on the likelihood of sexual transmission of genital herpes in prepubertal children is weak [25]. More information is needed on the prevalence of infections with HSV-1 and HSV-2 in children with and without a history of CSA to help with the interpretation of an infection in a child [24]. Most cases of primary HSV infections are asymptomatic or not clinically recognized. Prevalence must be estimated by measuring HSV-specific IgG. A total of 20–33% of US children from various socioeconomic strata are positive for IgG to HSV by 5 years of age. Among asymptomatic young adult women who have no history of any oral or genital lesions, over 50% have IgG to HSV-1 and over 10% have IgG to HSV-2 [4]. A HSV-prevalence study in the Netherlands reported that the sero-prevalence for HSV-type 1 is about 15% of the 1–4 year olds and 25–30% of the 5–9 year olds [30]. According to this data, our results do not differ from the general child population.

### Physical examination

The physical examinations in our cohort revealed no CSA-specific anogenital findings. This could be explained by the fact that most genital injuries in CSA heal with little or no residua, unless the injuries are severe. According to the perpetrator’s testimony, 13 children were sexually abused in 2010, and the abuse had taken place more than 1 year ago for all the other included children. When children are physically examined more than 72 h after the last abuse, physical signs specific for CSA are only found in a small minority of cases, 4 to 5% [2, 24].

In 8 children, CSA nonspecific anogenital findings, such as erythema, were reported. According to the RCPCH guideline, erythema is a nonspecific sign for CSA and the estimation of the degree of redness at physical examination is subjective. There are many other causes that need to be considered besides CSA as was discussed previously [24]. (Peri)anal erythema is seen in a small proportion of children with alleged CSA, but also in children selected for non-abuse [24].

### Behavior during physical examination

Yet, we found deviant behavioral responses to the physical examination in 28%. According to our experts, especially behavioral changes observed related to the anogenital examination were considered to be most worrisome.

The perception of non-abused children (boys and girls, 5–6 years of age) of the anogenital examination was examined by Gulla and colleagues (2007). They found that the anogenital examination was perceived (somewhat) negative by only 7.7% of the examined children and neutral positive by 92.2% (but significantly more distressing than examination of ears or mouth) [12]. However, according to parents and nurses, about one third of the children showed some distress in relation to the examinations; in the great majority of children, some minor symptoms of distress were expressed. In only 0.6% of the children, the nurses reported “a lot anxiety/restlessness” during the anogenital examination [12].

Several studies evaluating the distress perceived by children examined for alleged CSA [15, 19, 26, 27] are published. Marks and colleagues (2009) investigated the psychological stress in a sample of mainly girls and their caretakers before and after a physical examination. They found that two-third (66%) of the examined children felt scared before the exam. Significantly more stress was reported by older children (12 years and above). Children experienced less pain and felt less scared than they had anticipated, but the differences were not statistically significant. Though the negative comments of these children after the examination referred mainly to injections and blood tests rather than to the anogenital phase of the examination, they also found that caregiver knowledge of examination procedures was inversely associated with caregiver report of distress before the examination. This relationship was not observed for the children [19].

As opposed to the study from Marks and colleagues, other studies found that anogenital exams are not re-traumatizing or perceived as “strongly negative” [15, 27]. Anxious feelings prior to the examination seem to increase anxious behaviors during the examination [26].

In our cohort, 15 children (27.8%) showed deviant behavioral reactions related to the anogenital examination. Our percentage is much higher than found by Gulla and colleagues in non-sexually abused children [12], but relatively low in comparison with the experienced distress reported by Marks and colleagues in sexually abused children [19]. We do not know whether children or their parents experienced distress before, during, or after the examination. Our findings are solely based on observations during the examination by pediatricians and child behavioral specialists.

Literature shows that children who have some understanding of the examination report less symptoms of anxiety during the examination [26, 29]. Therefore, it is very important that the examination is carefully explained. Knowledge of preschool children’s perceptions of the anogenital examination and the best way to prepare these children is lacking.

### Strengths and limitations

Literature on physical signs and symptoms of CSA in boys of this age is scarce. This study presents unique data from a sample of 54, predominantly male, preschool confirmed victims of CSA. The level of evidence of CSA in these children was high. Data on the nature, frequency, and “timing” of the abuse were based on police reports including perpetrators’ confessions and decrypted pornographic images, though it is possible that the perpetrator was not 100% honest about the moment, nature, and frequency of the abuse.

There are also several limitations of this cohort study. First, a potential bias exists of both parents and clinicians who performed the evaluation. All parents became to understand that their child was a possible victim of CSA only shortly before their child’s examination at the OPD. Likely, they have been distressed and therefore parents either underestimated or overestimated problems in their child when interviewed. Consequently, they may have highlighted physical symptoms that would otherwise be considered normal or, conversely, they may not, as a result of the commotion, have been complete in their reports of physical symptoms. The fact that the included children were all abused by the same perpetrator introduces another potential bias concerning the STI-investigations and general interpretation of findings.

The clinicians involved in the evaluations were all experienced in evaluating suspected CSA, but never experienced a sexual abuse case of this extent. Considering the acute setting of the assessments at the OPD, there was no predefined study design, and consequently, data were not collected using standardized questionnaires but retrospectively from the medical records.

Urogenital and GI complaints were reported most often. Abdominal pain, anal or vaginal blood loss, and problems concerning urination or defecation were addressed by the pediatricians in all children because these were predefined topics on their topic list used for the semi-structured medical histories. Other complaints, such as for example neurologic complains (e.g., headaches), were not discussed semi-structured, and therefore, the outcomes are possibly biased. Nor were standardized criteria for scoring the physical complaints, such as the Rome III criteria for functional gastro-intestinal disorders, used. We do not know if all questions were asked to evaluate whether a child suffered from for example functional constipation.

Due to the absence of a control group in our study, we were only able to compare our findings to what is known from the literature. Therefore, we cannot comment on causality, based on our findings.

### Implications for future research and patient care

Considering the fact that findings at physical examination indicative for CSA are very rare in children, it is important to carefully observe the child’s behavior during the examination. We found that deviant behavior during anogenital examination might be a good indicator of CSA. Based on our findings, we would advise clinicians to focus specifically on behavioral observations during the physical examination. Especially sudden behavioral changes observed during the anogenital examination, such as anxiety, panic attacks, or shutting down, and “too easy and outgoing” reactions seem important. More research on children’s behavioral responses during anogenital examination is needed before we can make accurate recommendations. It is advised that during the physical examination, an observer is present to notice the child’s reactions, preferably a child behavioral specialist. Based on our current knowledge, children need to be prepared carefully and age adjusted before the physical examination, though further research is needed to investigate how young children can be prepared best.

As recommended by the RCPCH guideline, there is an urgent need for comparative primary research studies on CSA and anogenital injuries (in both female and male children) [24]. The shortage of studies reporting on an association between CSA and physical complaints in children is striking. We recommend that future research will focus on the association of CSA and functional somatic symptoms in young, preferably preschool, children. Ideally, a prospective cohort study investigating the association of CSA and functional somatic symptoms in young (preschool) children is performed.

## Conclusion

In our cohort of confirmed young CSA victims, the most prominent finding was a deviant behavioral reaction during physical examination, which was noted in about one third of the confirmed CSA victims. In children who experienced anal/vaginal penetration, significant more behavioral reactions were noted (47%) compared to children who did not. Physical complaints and physical signs at anogenital examinations were non-specific. Observation of the child’s behavior during the physical examination is important. The presence of a professional observer during the physical examination is recommended. Assessment of alleged CSA should be done systematically by experienced and capable experts with full knowledge of the scientific evidence on symptoms and whether or not these are related to CSA.

## Electronic supplementary material

ESM 1 (DOC 182 kb)

## Abbreviations

AMC: Academic Medical Center
CSA: Child sexual abuse
GI: Gastro-intestinal
STI: Sexually transmitted infection
OPD: Outpatient department
RCPCH: Royal College of Pediatrics and Child Health
