# Supplementary material for: Physical symptoms in very young children assessed for sexual abuse: a mixed method analysis from the ASAC study
Source: Eur J Pediatr. 2017 Aug 26;176(10):1365–74. doi: 10.1007/s00431-017-2996-7 (PMC5607905; doi:10.1007/s00431-017-2996-7)
Supplement: Supplementary file 1 — (DOC 182 kb) [file 431_2017_2996_MOESM1_ESM.doc]

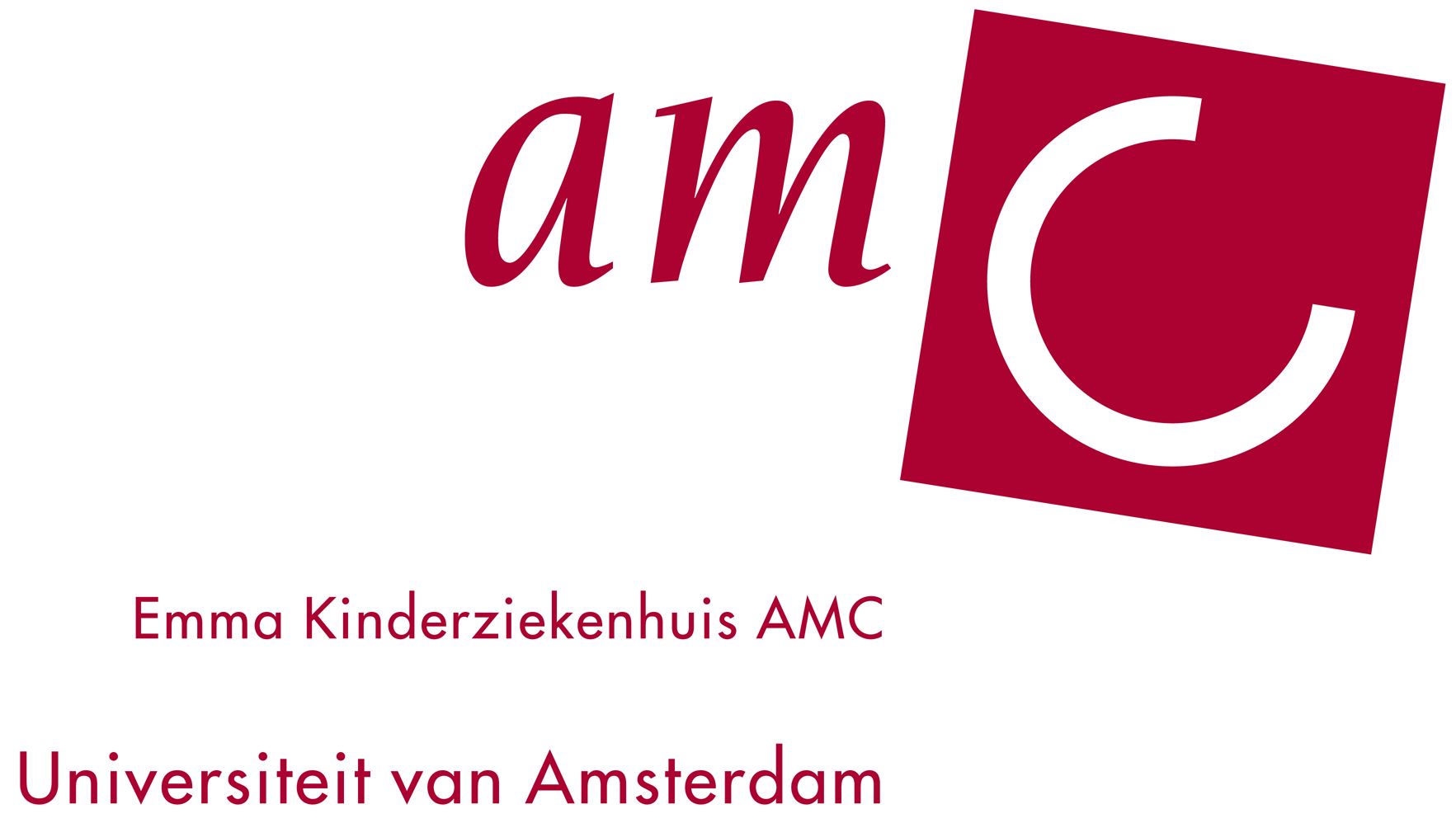
**Additional medical chart for suspected CSA**

Patient identification code:

**Name patient:**

**Name pediatrician:**

**Date:**

**I. Explanation Procedure**

First consult with pediatrician, social worker, child developmental specialist and forensic physician (+/- 10 min).

Interrogation by the police has taken place or has to take place. This visit is for medical consultation and to start aftercare.

Continuation consult with the child and performance of physical examination (next 20 min).

Discuss medical photographer with parents.

Discuss laboratory investigations.

Social worker talks with parents while the child is seen by the child specialist (about 30 min).

Telephone consult to discuss laboratory results.

*Discuss possible emotions*

We understand that/ it is understandable that everything happening at the moment arouses emotions. Unfortunately we have a strict schedule and therefore need to ask you to discuss those with the social worker at the end of the consult.

**II Other specific questions**

1. What did you tell your child(ren) about the OPD-visit?

2. In which period was your child exposed to the suspect?

3. Did you notice any abnormalities? If yes, what?

4. Specific complaints

- Abdominal pain: yes / no
- Eating problems: yes/no
- Anal or vaginal bloodloss noticed: yes/nee
- Problems concerning urination or defecation: yes/no
- Blisters mouth and genitals: yes/no
- Behavioral problems: yes/no
- Sleeping problems: yes/no
- Regression: yes/no
- Age inappropriate sexual behavior: yes/no
- Learning problems: yes/no
- Problems with social contact: yes/no

**III Child and parents/caretakers ouders/verzorgers**

1. Explanation procedure to the child
2. Explanation of physical examination (top to toe) including ano-genital area (use the terms the family is used to)
3. Short medical history (vaccination hepatitis B?)
4. Physical examination (top-to-toe)
5. Photographs anogenital area
6. Laboratory testing
   1. Set: last contact < 3 months ago
   2. Set: last contact > 3 months ago
   3. Set: last contact < 2 weeks ago

**IV Telephone consult about laboratory results**
